# Unveiling Cognitive Interference: fNIRS Insights Into Poststroke Aphasia During Stroop Tasks

**DOI:** 10.1155/np/1456201

**Published:** 2025-03-31

**Authors:** Chong Lu, Mingzhu Wang, Likan Zhan, Min Lu

**Affiliations:** ^1^Department of Rehabilitation Medicine, Tongji Hospital, Tongji Medical College, Huazhong University of Science and Technology, Wuhan, Hubei, China; ^2^College of International Education, Minzu University of China, 27 Zhongguancun South Avenue, Beijing 100081, China; ^3^Cognitive Science and Allied Health School, Beijing Language and Culture University, Beijing 100083, China; ^4^Institute of Life and Health Sciences, Beijing Language and Culture University, Beijing 100083, China; ^5^Key Laboratory of Language and Cognitive Science (Ministry of Education), Beijing, China

**Keywords:** aphasia, functional near-infrared spectroscopy, Stroop task

## Abstract

This study examined blood oxygenation changes during a modified Stroop task with colored Chinese words using functional near-infrared spectroscopy (fNIRS) in patients with poststroke aphasia. The task included three conditions: neutral, congruent, and incongruent. Participants consisted of 15 healthy adults and 15 patients with poststroke aphasia. Compared to healthy adults, aphasic patients showed significantly longer reaction times and reduced accuracy across all conditions, with a more pronounced interference effect in the incongruent condition. fNIRS analysis revealed distinct neurophysiological differences: decreased activation in Broca's area, increased activation in the ventromedial frontal pole, and atypical recruitment of the left dorsolateral prefrontal cortex (DLPFC) during Stroop interference tasks. These findings highlight the differing neural mechanisms underlying cognitive interference in poststroke aphasia. The integration of fNIRS with the Stroop task enhances our understanding of intentional inhibition deficits and the impact of cognitive interference in aphasic patients. Importantly, these results suggest that deficits in cognitive control and abnormalities in prefrontal regions, such as the frontal pole and DLPFC, may be potential targets for noninvasive neuromodulation to improve cognitive control in poststroke aphasia. The observed atypical activation patterns in these regions underscore their critical role in managing cognitive interference and intentional inhibition. Noninvasive brain modulation techniques may offer promising strategies for modulating these neural mechanisms. This study underscores the need for targeted interventions that address prefrontal dysfunctions and emphasizes the value of visual language tasks in exploring the complex relationship between language deficits and cognitive control in this population.

## 1. Introduction

Aphasia, a complex communication disorder often caused by stroke or other brain injuries, extends beyond language impairments to include various cognitive deficits [1, 2]. Understanding the interplay between these language and cognitive challenges is crucial for advancing rehabilitation strategies. Foundational cognitive functions, such as attention and intentional inhibition, are vital for language processing and daily activities [3, 4]. Deficits in these areas—such as reduced task-switching ability and sustained attention—can worsen communication difficulties and delay recovery [5, 6]. These impairments significantly affect overall functionality and quality of life. With the prevalence of poststroke aphasia ranging from 25% to 50% [7], it is essential to understand its cognitive and linguistic dimensions. This study innovates by combining Stroop tasks with functional near-infrared spectroscopy (fNIRS) to explore cognitive control deficits and the neural mechanisms underlying poststroke aphasia. Through this experimental design, we aim to deepen our understanding of neurocognitive deficits in aphasia, providing a theoretical basis for improving cognitive rehabilitation therapies. The study also seeks to advance the use of brain modulation technologies in aphasia treatment, focusing on selecting brain regions and developing modulation strategies. Additionally, by examining cognitive tasks within the Chinese cultural context, this research aims to support culturally sensitive rehabilitation strategies, particularly regarding Stroop interference effects and cognitive load in the Chinese visual language system.

The Stroop task, originally designed to assess interference control and intentional inhibition [8, 9], has become an important tool for exploring cognitive impairments in aphasia. It requires participants to name the ink color of incongruent color words, highlighting deficits in attentional control and inhibition. For individuals with poststroke aphasia, difficulties managing conflicting information or suppressing interference reveal cognitive control deficits crucial for effective communication [10]. Beyond language deficits, aphasic individuals often show impairments in higher-order cognitive functions such as attention and working memory [6, 11]. Adapting the Stroop task for Chinese-speaking populations introduces unique cognitive challenges. Due to the logographic nature of Chinese, tasks involving character recognition and color naming impose additional cognitive demands. Participants must inhibit automatic responses tied to complex character recognition while focusing on color identification [12, 13]. The visual complexity of Chinese characters requires more extensive processing than alphabetic scripts, potentially exacerbating interference effects. For bilingual speakers, research indicates greater within-language interference when responding in Chinese, compared to switching between languages, reflecting how language proficiency and experience influence cognitive control [14]. Event-related potential (ERP) studies have shown greater neural activation in incongruent trials involving Chinese characters, indicating the role of prefrontal cortex activation in conflict resolution [13]. Reduced ability to resolve interference, seen in longer response times or higher error rates in incongruent conditions, directly correlates with deficits in cognitive control mechanisms critical for effective communication. Intentional inhibition—the active suppression of prepotent responses—is particularly impaired in aphasia, making it harder to filter irrelevant stimuli, manage interference, and sustain attention [15]. These impairments significantly hinder performance on tasks requiring selective attention, such as the Stroop task [16]. Idiom comprehension deficits in aphasia further illustrate these challenges, as individuals struggle to inhibit literal interpretations and process abstract meanings, highlighting the broader impact of cognitive interference on language processing [17]. In conclusion, the Stroop task offers a robust framework for assessing the interplay between language and cognitive processes in aphasia. By uncovering deficits in attention, inhibition, and cognitive flexibility, it provides valuable insights for clinical practice, informing the development of culturally relevant interventions and enhancing our understanding of aphasia's neurocognitive underpinnings.

The construct of cognitive interference in Chinese language Stroop tasks remains underexplored. As a logographic language, the Chinese Stroop task requires participants to inhibit automatic responses related to recognizing and reading characters while focusing on color identification. This task demands heightened cognitive control due to the simultaneous involvement of semantic and visual processing mechanisms. The cognitive load in processing Chinese characters is higher than in alphabetic languages, as it requires not just phonetic decoding but also complex character recognition and meaning retrieval [13]. This could lead to increased interference effects compared to adaptations of the Stroop task in alphabetic languages. Intentional inhibition plays a crucial role here. In aphasia, deficits in intentional inhibition are evident, making it challenging to filter irrelevant stimuli, manage interference, and sustain attention [15]. These impairments significantly affect an individual's ability to focus on tasks, filter distractions, and distinguish between relevant and irrelevant information. Research shows that people with aphasia struggle to manage interference from competing stimuli, which impedes their performance on tasks requiring selective attention, such as the Stroop task [16]. Additionally, studies on idiom comprehension deficits in aphasia further highlight the role of cognitive interference. Aphasic individuals may struggle to inhibit literal meanings and recognize figurative interpretations, demonstrating the broader challenges in language processing caused by cognitive interference [17]. These impairments reflect deeper issues in higher-order cognitive functions, which are essential for both language processing and effective communication. This approach lays the groundwork for cross-linguistic and culturally sensitive cognitive assessments, which are crucial for refining therapeutic methods across different language groups and improving clinical outcomes for those affected by aphasia.

fNIRS has emerged as a powerful neuroimaging tool for investigating neurocognitive deficits, offering several advantages over traditional methods like functional magnetic resonance imaging (fMRI). Unlike fMRI, fNIRS enables real-time, noninvasive monitoring of cortical activation using near-infrared light (650–900 nm) to measure changes in oxyhemoglobin (HbO), deoxyhemoglobin (DHb), and total hemoglobin (tHb). This portability and resistance to motion artifacts make fNIRS especially suitable for populations with limited mobility, such as stroke survivors. The ability to dynamically assess brain activity during task performance is invaluable for studying cognitive processes like attention, inhibition, and executive functions. These strengths have positioned fNIRS as a leading tool in research on the neural basis of language and cognition [18, 19]. Beyond its technical strengths, fNIRS has proven useful in clinical populations. Its integration with cognitive tasks like the Stroop test has provided valuable insights into cognitive interference. The Stroop task, which requires participants to manage conflicting information by naming the ink color of incongruent words, is a well-established method for studying attentional control and intentional inhibition. fNIRS studies have shown that younger adults exhibit stronger hemodynamic responses in the dorsolateral prefrontal cortex (DLPFC) during incongruent trials compared to older adults [20]. Similarly, individuals ADHD, or traumatic brain injuries show altered DLPFC activation during Stroop tasks, reflecting impairments in neurovascular coupling and cognitive interference management [21, 22]. These findings underscore the DLPFC's crucial role in resolving cognitive conflicts and highlight the value of fNIRS in identifying functional impairments in various clinical conditions. In poststroke aphasia, where language processing and executive functions are often compromised, fNIRS has been instrumental in uncovering underlying neural mechanisms. Research shows that aphasic patients often exhibit atypical activation patterns during language tasks, with increased activity in nondominant brain regions and reduced connectivity in typical language networks [23, 24]. These disruptions are compounded by deficits in attention and intentional inhibition, which are essential for effective language comprehension and production. By combining fNIRS with the Stroop task, researchers can examine how aphasic individuals manage cognitive interference and assess the neural correlates of impaired inhibitory control. Preliminary studies suggest that aphasic patients show unique hemodynamic responses during tasks requiring intentional inhibition, such as naming colors while suppressing automatic reading tendencies. For example, abnormal activation in regions like Broca's area and the supplementary motor cortex has been linked to challenges in language processing under interference conditions [19, 25]. These findings align with broader research on attentional deficits and impaired executive functions in language recovery. Integrating fNIRS with the Stroop test not only allows for real-time monitoring of these processes but also provides a strong framework for evaluating rehabilitation strategies tailored to aphasic patients' specific needs. This study builds on this foundation by using fNIRS to investigate intentional inhibition under Stroop interference in poststroke aphasia patients. By focusing on hemodynamic response changes during task performance, the research aims to uncover the neural mechanisms underlying cognitive deficits in this population.

This study investigates cognitive processing deficits in poststroke aphasia patients using fNIRS and the Stroop task. The classification of aphasia follows the Western Aphasia Battery (WAB), a standard diagnostic tool. We hypothesize that aphasic patients will show prolonged response times, reduced accuracy, and abnormal prefrontal activation patterns compared to healthy controls, reflecting impairments in executive control mechanisms, particularly in the DLPFC. Previous studies have shown the utility of the Stroop task in assessing cognitive control deficits, with elderly individuals exhibiting bilateral DLPFC activation [26] and stroke patients showing inverse activation patterns in the prefrontal cortex [27]. This research focuses on the cognitive and neural characteristics of aphasia in a Chinese population, aiming to provide insights into the relationship between behavioral deficits and neural mechanisms. By combining fNIRS with the Stroop task, we offer a new approach to studying aphasia, with potential implications for rehabilitation interventions.

## 2. Materials and Methods

### 2.1. Subjects

A total of 15 healthy adults (11 males and 4 females) and 15 patients with poststroke aphasia (10 males and 5 females) participated in this study. Despite the relatively small sample size (*n* = 15 per group), we leveraged power analysis to ensure the adequacy of our design. Previous fNIRS studies, such as those examining Stroop task performance in related populations, have reported significant effects, with effect sizes ranging from Cohen's *d* = 0.8 to 1.0 [28]. Thus, we determined that our sample size would be appropriate to detect large effects. Using G^*⁣*^*∗*^^Power, we confirmed that a sample size of 15 participants per group would provide 80% power to detect an effect size of ~0.8.

Healthy subjects were included based on the following criteria: aged 18–85, native Mandarin speakers, with a minimum of an elementary school education, and no history of vision or hearing impairment or significant neurological disorders. Aphasic patients were included if they were aged 18–85, had experienced a first-time stroke confirmed by CT or MRI, had stable vital signs, and demonstrated aphasia as diagnosed by experienced speech–language pathologists at Tongji Hospital's Rehabilitation Medicine Department. Aphasia was assessed and confirmed using the Chinese version of the WAB. Patients with significant comprehension or command execution deficits were excluded, ensuring that task demands would be manageable. Furthermore, cognitive function was evaluated using the MoCA scales, and those with moderate to severe cognitive impairments were excluded. All subjects completed the color comprehension tasks from the Chinese version of the WAB before the formal experiment, and only those achieving 100% accuracy were allowed to participate, ensuring reliable performance on the Stroop task. The study was approved by the Institutional Ethical Committee of Tongji Hospital (ID: TJ-IRB202404052). Ethical compliance was rigorously maintained, and all participants provided informed consent before participating. The control group was composed primarily of spouses or family members of aphasic patients, ensuring demographic comparability between groups. Detailed demographic information is presented in Table 1.

### 2.2. Psychophysical Procedures

**Figure 1:**
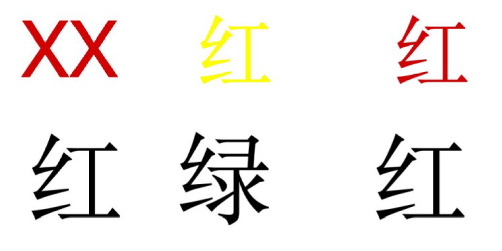
Here is an example of a single trial in the color–word matching Stroop task for neutral, incongruent, and congruent conditions. If the top word's color matches the bottom word's meaning, then the correct response would be “yes”.

We adopted a paradigm from previous research [28] and modified the stimulus materials into Chinese. The Stroop task was presented in an event-related ways. Two stimuli were presented on two separate lines on the screen, and the subjects were instructed to make a judgment test on whether the bottom line's Chinese characters matched the color of the top line (Figure 1). Three experimental conditions were manipulated. In all three conditions, the bottom line was always a color character printed in black, including the second line in Figure 1. The critical difference between the three conditions lies in the top line. In the neutral condition, the neutral symbol XX was printed on the top line with a color that is the same as the meaning of the Chinese character printed in the second line, including the left column of Figure 1. In the congruent condition, the meaning and the color of the top character are the same. For example, if the top character is “红” (red), it will also be printed in red color. In contrast, in the incongruent condition, the meaning and the color of the top character differ, which results in Stroop interference. This misalignment between the semantic meaning and the color of the top character generates cognitive conflict, as the brain experiences difficulty processing conflicting information. In each condition, half of the trials presented the top stimulus color, which matched the semantic color of the bottom Chinese character (Figure 1). The other half displayed a mismatch between the two, requiring participants to judge whether the top stimulus color corresponded to the meaning of the bottom character. Responses were recorded via a key press, with “1” indicating “yes” and “2” indicating “no.”

The experimental program was presented using E-Prime software and connected to a near-infrared device with 53 channels. There were 30 trials in one experimental run (10 neutral trials, 10 incongruent trials, and 10 congruent trials) presented randomly with a 12-s interstimulus interval. With a maximum stimulus duration of 4 s, the word stimuli remained on the screen until the response was made. The screen was blank between trials. To ensure consistency and reliability of the experiment, the task parameters were not adjusted as these parameters have been widely validated and proven effective in previous studies [27, 28]. Before the task began, aphasic participants were provided with a targeted demonstration using a PowerPoint presentation to ensure they accurately understood the task. Only after confirming their comprehension were they included in the experiment. During the WAB assessment, it was ensured that all aphasic participants were able to correctly recognize and identify colors. The experimental conditions were consistent for all participants, with stimuli presented randomly due to the event-related design. Participants were given sufficient time to respond within the maximum stimulus duration of 4 s, with a 12-s interstimulus interval between trials. This timing was considered ample for aphasic participants to process and respond to the stimuli.

### 2.3. Data Acquisition by fNIRS

**Figure 2:**
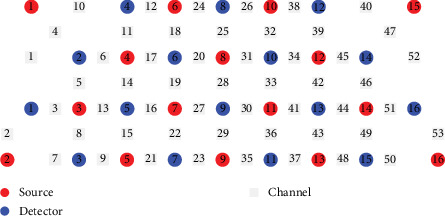
Optode arrangement and the entire device cover the prefrontal and temporal lobes of the brain.

A 53-channel fNIRS device (Wuhan Znion Technology Co., Ltd., China) was used to acquire data during the Stroop task. The device's detailed specifications are consistent with those described in previous study [29, 30]. The system employs 16 emitter–detector pairs to measure changes in HbO, DHb, and tHb concentrations. The device operates at wavelengths of 760 and 850 nm with a sampling frequency of 10 Hz. The distance between each emitter and detector probe is ~3 cm, and each pair of probes defines a measurement channel (Figure 2). The placement of the optodes was guided by the international 10–20 system of electrode placement, which is commonly used in EEG and fNIRS studies. This system ensures accurate and standardized positioning of the probes relative to the underlying brain regions. Specifically, the lowest channels were positioned at Fp1 and Fp2, aligning with the 10–20 system's specifications. The optodes were positioned on the scalp of the forehead. The alignment of brain regions with measurement channels was based on established virtual alignment techniques, as described in prior literature [29, 30]. For greater transparency and reproducibility, please refer to the supporting information report for detailed MNI coordinates for each measurement channel. This additional material will help readers accurately interpret the brain regions associated with each channel and enhance the methodological rigor of the study. This method ensures that the regions of interest (ROI), such as the DLPFC and Broca's area, are accurately represented in the fNIRS data. The approach maintains consistency with previous studies and allows for reliable interpretation of the recorded data. The ROI included Broca's Area (channel 50), the Dorsolateral Prefrontal Cortex (CHannels 32 and 34), and the Frontal Lobe (channel 36).

### 2.4. Data Processing

Data preprocessing was performed using MATLAB (R2021b, MathWorks Inc.) with the Homer2 fNIRS MATLAB toolboxes, widely recognized for their effectiveness in motion artifact removal and data preprocessing [31, 32]. The raw fNIRS intensity data were first processed using the hmrIntensity2OD function to convert them into optical density. Motion artifacts were detected and corrected using hmrMotionArtifactByChannel, with the following parameters: tMotion set to 0.5, tMask set to 3.0, STDEVthresh set to 20.0, and AMPthresh set to 5.0. To further correct for motion, the hmrMotionCorrectSpline function was applied with a smoothing factor of 0.99. A decision was made to enable the turnon parameter to 1 to ensure proper function during processing. Following motion artifact correction, the data were band-pass filtered using the hmrBandpassFilt function, with a high-pass filter (HPF) of 0.010 Hz and a low-pass filter (LPF) of 0.10 Hz to eliminate baseline drift and physiological noise. Concentration changes in HbO and DHb were calculated using the hmrOD2Conc function, with a prespecified pathlength factor (ppf) of 6.0 for both HbO and DHb. Finally, the hmrBlockAvg function was applied to average the data within a time window defined by the range from −2.0 to 12.0 s relative to stimulus onset.

### 2.5. Data Statistics

The variance was analyzed using repeated measures of analysis of variance (ANOVA). The dependent variables were reaction time, accuracy, and changes in HbO, DHb, and tHb concentration in specific ROI. The within-subject factor in the ANOVA analysis was “stimulus condition: neutral/congruent/incongruent,” and the between-subject factor was “aphasia group/healthy control group.” Statistical Package for Social Sciences software (version 26.0) was used to perform the statistical analysis. Effect sizes were reported using partial eta squared (*η*^2^).

## 3. Results

### 3.1. Behavioral Results

**Figure 3:**
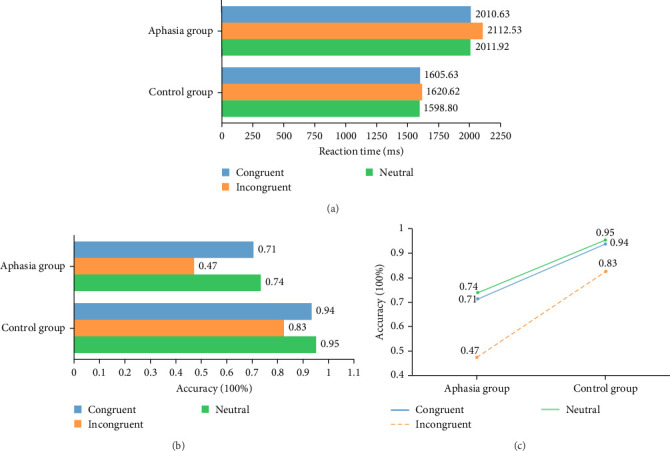
(a, b) All behavioral differences of the Stroop test. (c) The accuracy of the aphasia group and control group.

Based on reaction time, there was a significant difference between the groups according to the ANOVA (*F* (1, 28) = 16.050, *p* < 0.001), which indicated that a main effect exists and the groups exhibited. In the neutral stimulus condition, the aphasia group exhibited a longer reaction time than the control group (2011.92 ± 479.20 ms versus 1598.80 ± 340.36 ms; *F* (1, 28) = 5.434, *p*=0.03, partial *η*^2^ = 0.214). In the congruent stimulus condition, the aphasia group exhibited a longer response time than the control group (2010.63 ± 508.58 ms versus 1605.63 ± 306.55 ms; *F* (1, 28) = 5.117, *p*=0.035, partial *η*^2^ = 0.204). In the incongruent stimulus condition, the aphasia group exhibited a longer response time than the control group (2112.53 ± 622.49 ms versus 1620.62 ± 356.74 ms; *F* (1, 28) = 5.171, *p*=0.034, partial *η*^2^ = 0.205) (Figure 3a). The conditions were not significantly difference (*F* (2, 56) = 0.130, *p*=0.878 > 0.05), which indicated that it did not produce a differential relationship on reaction time. The lack of a significant difference between the group and condition (*F* (2, 56) = 0.063, *p*=0.939 > 0.05) indicates the absence of a second-order effect.

Based on the accuracy rate, ANOVA showed a significant difference between the groups (*F* (1, 28) = 47.862, *p* < 0.001), which indicated that a main effect exists and the groups exhibited a differential relationship. In the neutral stimulus condition, the aphasia group exhibited lower accuracy than the control group (0.74 ± 0.23 versus 0.95 ± 0.07; *F* (1, 28) = 8.834, *p*=0.008, partial *η*^2^ = 0.306). In the congruent stimulus condition, the aphasia group exhibited lower accuracy than the control group (0.71 ± 0.16 versus 0.94 ± 0.08; *F* (*1*, *28*) = 16.984, *p*=0.001, partial *η*^2^ = 0.459). In the incongruent stimulus condition, the accuracy of the aphasia group was lower than that of the control group (0.47 ± 0.22 versus 0.83 ± 0.08; *F* (1, 28) = 25.435, *p* < 0.001, partial *η*^2^ = 0.560) (Figure 3b).

The conditions were significantly different (*F* (2, 56) = 10.253, *p* < 0.001), which indicated that a main effect exists and the conditions produce a differential relationship on accuracy. In neutral and congruent conditions, there was no significant difference in accuracy between the two groups of subjects (*F* (1, 58) = 0.162, *p*=0.689, partial *η*^2^ = 0.004). Both groups of subjects exhibited significantly lower accuracy in the incongruent condition than in the congruent condition (*F* (1, 58) = 7.436, *p*=0.009, partial *η*^2^ = 0.150). Both groups of subjects exhibited significantly lower accuracy in the incongruent condition than in the neutral condition (*F* (1, 58) ≥ 8.447, *p*=0.006, partial *η*^2^ = 0.167) (Figure 3c). The groups and conditions did not differ significantly (*F* (2, 56) = 1.304, *p*=0.279 > 0.05), indicating the absence of a second-order effect.

### 3.2. fNIRS Results

Tables 2–4 present the ANOVA results for chromophore levels (HbO, DHb, and tHb) measured across different channels (34, 32, 36, and 50) under three experimental conditions: neutral, congruent, and incongruent. The tables include the mean values (± standard deviation, units in µmol·mm) for both the aphasia and control groups, along with the corresponding *F*-statistics, *p*-values, and partial eta squared (*η*^2^). Significant differences are highlighted, with specific channels showing notable variations. For a more detailed interpretation of the results, please refer to the supporting information.

**Figure 4:**
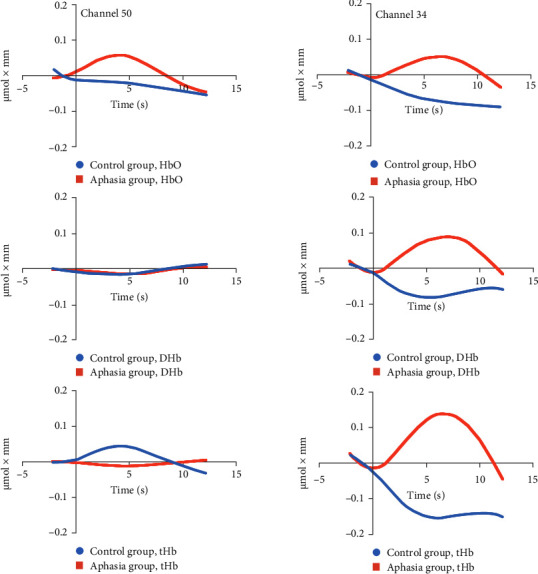
The time courses for chromophore in the incongruent condition of groups during the Stroop task of incongruent condition at channels 50 and 34. Average across all subjects. The *x*-axis represents time from 2 s before the onset of the Stroop task to 12 s after the task onset. The *y*-axis represents the change in hemoglobin concentration, with values ranging from −0.2 to 0.2 µmol·mm.

Figure 4 provides time series plots of chromophore levels (HbO, DHb, and tHb) across the key channels—Broca's area (50) and the left DLPFC (34)—for both the aphasia and control groups. These plots illustrate the trends in brain activity under neutral, congruent, and incongruent conditions, aligning with the significant differences reported in Table 4.

Under the incongruent condition, compared to the congruent and neutral conditions, the control group showed a trend of decreased HbO, DHb, and tHb levels in channel 34 (left DLPFC). This was accompanied by an increase in reaction time and a significant decrease in accuracy, suggesting that while the control group demonstrates relatively efficient cognitive conflict control abilities, additional time and cognitive resources are needed when facing complex semantic conflicts. This indicates that the DLPFC may adjust blood oxygen metabolism to optimize conflict control. In channel 32 (left DLPFC), the control group exhibited higher blood oxygen levels under the incongruent condition, which likely supported better performance on the conflict task, maintaining relatively high accuracy. However, in channel 36 (frontal pole), although there were no significant changes in HbO or tHb, the trend suggested that the control group did not rely heavily on the frontal pole for high-load tasks, indicating a limited role in routine conflict processing. In channel 50 (Broca's area), the control group displayed higher HbO levels, reflecting the efficient activation of this region for language processing and semantic integration, which helped manage complex semantic conflicts. In contrast, the aphasia group showed different neurophysiological and behavioral patterns. In channel 34 (DLPFC), the aphasia group exhibited an increase in HbO, DHb, and tHb levels, suggesting that more neural resources were mobilized to manage the high cognitive load. However, this increased activation did not improve behavioral performance, as evidenced by longer reaction times and significantly lower accuracy. This suggests that the DLPFC in the aphasia group operates with lower functional efficiency and requires compensatory activation. In channel 32 (left DLPFC), the aphasia group demonstrated significantly lower HbO and tHb levels, particularly under the incongruent condition, indicating that they were unable to effectively engage this region for conflict resolution. This pattern was consistent with poorer behavioral performance, including lower accuracy and longer reaction times. In channel 36 (frontal pole), the aphasia group exhibited significantly higher DHb levels compared to the control group, indicating the recruitment of additional resources for compensatory processing. However, this did not significantly improve their behavioral performance. Finally, in channel 50 (Broca's area), the aphasia group showed significantly lower HbO levels, indicating impaired language and semantic processing abilities, which likely contributed to their extended reaction times and reduced accuracy in the conflict task. In summary, the neurophysiological differences between the control group and the aphasia group highlight distinct mechanisms of cognitive conflict processing. The control group efficiently modulates neural activity in key regions like the DLPFC and Broca's area to manage semantic conflicts, while the aphasia group shows inefficient resource allocation and lower activation in these regions, resulting in impaired performance. Despite compensatory activation in certain areas, these mechanisms do not fully restore the aphasia group's cognitive abilities, revealing significant challenges in managing complex tasks.

## 4. Discussion

This study replicated the experimental paradigm from previous research [28] and extended it by applying it to a group of patients with aphasia. Using fNIRS, we examined blood flow dynamics in aphasic patients compared to healthy controls during a Stroop color–word interference task. Our findings revealed significant hemodynamic differences between the aphasia and control groups across several brain regions when processing incongruent color–word stimuli. Specifically, aphasic patients demonstrated reduced accuracy and prolonged reaction times under incongruent conditions compared to controls. fNIRS data showed anomalous hemoglobin concentration changes (HbO, DHb, and tHb) in the left DLPFC, with significant DHb increases in the ventromedial frontal pole and reduced HbO levels in Broca's area relative to controls. These results highlight distinct hemodynamic response patterns in aphasia during interference-based cognitive tasks. This research provides valuable insights into the neural mechanisms underlying cognitive impairments in aphasia and underscores the utility of fNIRS in advancing our understanding of these deficits. Such findings are critical for guiding targeted therapeutic strategies aimed at alleviating cognitive deficits in poststroke aphasia. Furthermore, altered hemoglobin concentration patterns in the left DLPFC emerge as potential neuroimaging markers, reflecting deficits in attention and intentional inhibition. Atypical hemodynamic responses, particularly reduced or disrupted activation in the left DLPFC, suggest impaired cognitive control mechanisms in individuals with aphasia.

Our study used a Chinese color–word Stroop task to examine the effects of group (aphasia vs. control) and stimulus conditions (neutral, congruent, and incongruent) on reaction time and accuracy. The results demonstrated significant main effects of group, with aphasic individuals showing prolonged reaction times and reduced accuracy across all conditions. This finding supports prior research on aphasia using the verbal Stroop task [15]. The slower reaction times observed in the aphasia group indicate impairments in cognitive processing speed and response initiation across various task demands. Additionally, the significant main effect of group on accuracy reflects persistent difficulties in correctly identifying and responding to color–word stimuli. These challenges likely stem from deficits in attentional control, inhibitory processes, and language integration, compounded by a slower rate of language processing that delays the integration of visual and linguistic information. This interpretation aligns with previous studies highlighting impairments in cognitive control, executive function, and language processing speed in aphasia [33, 34]. Furthermore, performance declined notably in the incongruent condition compared to the congruent and neutral conditions, consistent with the Stroop effect, which arises from conflicting semantic and color information [15, 28, 35]. The observed interference effects disrupted accurate judgments in both groups, illustrating the robustness of the Stroop task in uncovering cognitive deficits associated with aphasia. These findings deepen our understanding of the impact of aphasia on attentional allocation, inhibitory control, and linguistic integration, emphasizing deficits in visual–language cognitive processing at a fundamental level in this population.

The fNIRS results revealed distinct neurophysiological patterns in aphasic individuals compared to controls under different experimental conditions. In the neutral condition, reduced HbO levels in Broca's area among individuals with aphasia suggest altered baseline metabolic activity or oxygen utilization in this critical language region. This finding aligns with literature reporting structural and functional abnormalities in Broca's area in aphasic patients, which likely contribute to deficits in language production and comprehension [36, 37]. During the congruent condition, elevated DHb levels in the ventromedial frontal pole of aphasic participants suggest increased neural activation or oxygen extraction in this region compared to controls. This pattern may reflect compensatory neural mechanisms or inefficiencies in processing semantic and cognitive control tasks [38, 39]. These results underscore the complexity of neural responses in aphasia and highlight the heterogeneous nature of brain activation during cognitive tasks, particularly in regions associated with language and executive function. Significant differences in HbO, DHb, and tHb concentrations were observed between aphasic individuals and controls during the incongruent condition of the Stroop task, reflecting distinct hemodynamic responses across various brain regions. Specifically, the left DLPFC exhibited a unique pattern in aphasic individuals, characterized by complementary changes in HbO, DHb, and tHb levels compared to controls. The differing activation patterns observed in channels 32 and 34, both within the left DLPFC, are particularly intriguing. One plausible explanation for these opposing patterns is spatial variability in neural activation within the DLPFC. Channel 34 may capture a more robust or localized increase in neural activity, while channel 32 may reflect reduced or altered activation in a nearby subregion. This could represent a shift in the focus or efficiency of activation in the left DLPFC among individuals with aphasia. For instance, the increased HbO, DHb, and tHb levels in channel 34 might indicate a compensatory response or heightened metabolic demand, while the decreased values in channel 32 could reflect impaired neuronal activation or disrupted hemodynamics in an adjacent subregion. Another explanation may involve neural reorganization in response to aphasia [40]. In many cases, aphasia triggers compensatory mechanisms, with some brain regions adapting to the loss of functional integrity. These adaptations may result in differential activation patterns within closely situated areas. The observed discrepancies may reflect broader changes in local cortical circuits, where certain subregions become hyperactive to compensate for deficits while others show reduced activation due to inefficiencies or disuse. The differential activation across neighboring channels may thus indicate complex region-specific adjustments to aphasia. Alterations in DLPFC activity could lead to localized increases in activity in some areas, coupled with decreases in others, as part of broader network reorganization [41]. These patterns may arise from the brain's attempts to adapt to functional losses by redistributing cognitive processing demands or modifying connectivity patterns. Such variations are reflected in changes to HbO, DHb, and tHb levels, contributing to the observed discrepancies in hemodynamic response. Reduced HbO levels in Broca's area among aphasic individuals further underscore compromised neural activity in this critical language region. This finding may indicate disrupted neural mechanisms shared between Broca's and Wernicke's areas [42], essential for language comprehension and production. The diminished HbO levels in Broca's area suggest reduced oxygen extraction or metabolic demand, potentially reflecting inefficient neural processing during cognitive tasks involving semantic and attentional control. These observations provide important insights into the neurobiological underpinnings of aphasia, emphasizing the interplay between hemodynamic responses and cognitive deficits. The findings also suggest that individuals with aphasia employ alternative neural strategies, likely involving compensatory mechanisms, to manage cognitive challenges compared to neurotypical individuals.

Behavioral and fNIRS results revealed significant differences between the control and aphasia groups in processing cognitive conflict and allocating neural resources. The control group exhibited efficient conflict management, reflected by decreased HbO, DHb, and tHb levels in the left DLPFC under incongruent conditions, alongside better behavioral performance. The reduction in oxygenation across the DLPFC and Broca's area in the control group suggests an adaptive neural response to the cognitive demands of the task. This efficient processing likely highlights the DLPFC's role in interference control [43] by optimizing resource allocation. Additionally, higher HbO levels in Broca's area in the control group support its involvement in language processing and semantic integration [44] during conflict resolution. Conversely, the aphasia group demonstrated increased HbO, DHb, and tHb levels in channel 34 of the DLPFC, indicating a greater demand for neural resources to manage the task. However, this compensatory activation did not translate to improved performance, as evidenced by their prolonged reaction times and lower accuracy. This inefficiency may stem from disruptions in the DLPFC's ability to dynamically allocate resources, compounded by language deficits characteristic of aphasia. These impairments likely affect both executive function and language processing, further hindering task performance. In channel 32 (left DLPFC), the aphasia group exhibited significantly lower HbO and tHb levels, reflecting insufficient engagement of regions critical for cognitive control. This aligns with their poor task performance, as they struggled to recruit adequate neural resources to resolve semantic conflict. Increased DHb levels in channel 36 (frontal pole) suggest reliance on compensatory activation, though this strategy was inadequate for overcoming task-related challenges. Moreover, significantly lower HbO levels in channel 50 (Broca's area) underscore a critical difficulty in language and semantic processing. Broca's area, essential for language production and comprehension [45, 46], showed reduced activation, likely contributing to the aphasia group's increased cognitive load and behavioral deficits. In summary, the aphasia group demonstrated neural resource allocation inefficiencies, particularly in the DLPFC and Broca's area, leading to impaired task performance and conflict resolution. Although some compensatory activation was observed, it was insufficient to overcome deficits in cognitive control and language processing. These findings underscore the need for targeted rehabilitation strategies to address disruptions in critical brain regions and improve outcomes in aphasia patients.

Under incongruent interference conditions, the abnormal blood flow in the left frontal lobe observed in poststroke aphasic patients aligns with prior findings from Stroop studies on stroke populations, emphasizing shared neural disruptions linked to stroke-related impairments. Research has demonstrated that, in healthy individuals, the Stroop task activates the DLPFC, highlighting its role in cognitive control [28, 43]. In acute ischemic stroke (AIS) patients, fNIRS studies have identified reduced activation patterns in the prefrontal cortex, correlating with impaired executive functions [27]. For post-ischemic stroke executive impairment (PISEI), fNIRS has further shown abnormal enhancements in the functionality of the left prefrontal and motor cortices during task execution. Notably, significant cognitive improvements were observed following transcranial magnetic stimulation (TMS) therapy, accompanied by enhanced functional activity in related brain regions [23]. Similarly, in traumatic brain injury (TBI) patients, fNIRS studies revealed abnormal increases in frontal lobe activity during Stroop task performance, highlighting inefficiencies in processing cognitive challenges [44]. Together, these findings underscore the value of combining the Stroop task with fNIRS to assess neurological disorders, providing a robust neurobiological foundation for personalized therapeutic strategies and rehabilitation programs. Abnormal frontal lobe blood flow in aphasia highlights the prefrontal cortex's critical role in cognitive control and intentional inhibition functions. Aphasic patients often experience difficulties in language expression and comprehension, which directly affect Stroop task performance. The Stroop task requires participants to quickly and accurately identify the color of words while ignoring their semantic meaning. However, aphasic individuals face increased interference and confusion when processing both color and semantic information due to language impairments [15], resulting in notable performance deficits compared to healthy controls. fNIRS technology enables real-time monitoring of brain activity in aphasic patients during Stroop task execution, particularly in the left prefrontal cortex and language-related areas, offering valuable insights into their neurocognitive functioning.

The aberrant blood flow in the left DLPFC of individuals with aphasia provides further support for the conflict monitoring theory, which posits that conflicts arise from inconsistencies or competition between cognitive information or behavioral responses [47, 48]. Such conflicts can span various cognitive dimensions, including meaning, color, and shape [49]. The Stroop task, a well-established conflict paradigm [50], exemplifies this by requiring participants to identify word color while ignoring its semantic meaning, inducing competition between automatic reading and instructed color naming [26, 51]. The left DLPFC plays a pivotal role in conflict processing and cognitive control, with its activity closely linked to efficient conflict resolution and behavioral performance during tasks such as the Stroop task [52, 53]. This region helps suppress inappropriate responses and mitigate interference by downregulating conflicting signals and enhancing inhibitory ones, thus enabling more effective conflict processing [53, 54]. Furthermore, the DLPFC forms part of a broader network, including the anterior cingulate cortex, striatum, and inferior frontal gyrus, which collectively supports conflict detection and executive control [55, 56]. Damage or dysfunction within this network, as observed in individuals with aphasia, can lead to attentional control difficulties and increased susceptibility to interference effects during tasks like the Stroop task. Cognitive control theory underscores the broader importance of the DLPFC in executive functions, including goal setting, inhibition, working memory, and interference control [57, 58]. In aphasic individuals, deficits in these areas may manifest as difficulties in suppressing irrelevant semantic interference and maintaining attentional focus, both of which are crucial for accurate task performance [59]. The language processing deficits observed in this study during Stroop tasks suggest impaired cognitive control in aphasia. Understanding these neural mechanisms not only provides insights into cognitive impairments associated with aphasia but also paves the way for developing targeted interventions to enhance both language and executive control abilities in affected individuals.

This study addresses a critical gap in existing research by exploring how cognitive control rehabilitation techniques, commonly applied in general poststroke recovery, can be tailored for individuals with poststroke aphasia. Previous Stroop task studies have demonstrated that transcranial direct current stimulation (tDCS) targeting the DLPFC reduces errors by enhancing sustained attention [60], while TMS improves cognitive control and task performance in both healthy individuals and stroke patients [23, 61]. These findings highlight the potential of neuromodulation techniques for cognitive rehabilitation. The rationale for using rTMS and tDCS in stroke rehabilitation is their capacity to modulate brain activity and connectivity, as evidenced by fNIRS metrics [23]. rTMS induces changes in cortical excitability and connectivity, while tDCS alters neuronal membrane potentials, influencing cognitive processes and neural activation patterns. Both techniques have shown effectiveness in improving cognitive deficits by targeting specific brain regions associated with impaired functions [62, 63]. Building on this foundation, our research expands these interventions to cognitive control rehabilitation for poststroke aphasia, focusing on the prefrontal cortex—identified in our study as exhibiting abnormal activity in aphasic patients. We hypothesize that neuromodulation targeting these regions could enhance cognitive control and language processing in this population. To assess the efficacy of these interventions, we propose combining Stroop tasks and fNIRS. The Stroop task will evaluate cognitive control and processing speed, while fNIRS will track changes in cerebral hemodynamic responses. Through longitudinal efficacy assessments at multiple time points, we aim to monitor how rTMS and tDCS influence hemodynamics and cognitive performance in aphasia patients. Neurophysiological techniques such as EEG and fMRI have provided deeper insights into the Stroop task's neural mechanisms. For example, EEG studies have shown that Stroop effects involve multiple stages of cognitive control with distinct temporal dynamics [64]. fMRI research has highlighted an excitatory–inhibitory network involving the frontal cortex and cerebellum [65], while resting-state fMRI studies have linked prefrontal activity to individual Stroop performance [66]. Additionally, studies indicate that increased working memory load impairs Stroop performance due to resource competition [67], and the anterior cingulate cortex plays a central role in conflict resolution [68]. Although these findings primarily stem from healthy adults, the application of Stroop tasks to aphasic individuals remains underexplored. To better understand the interplay between cognitive control and language deficits, future research should incorporate multimodal approaches, such as combining EEG and fMRI, to investigate brain activity in individuals with aphasia. Our study, by integrating the Stroop task with fNIRS, fills an important gap in aphasia research. This novel approach extends beyond prior behavioral studies [15] to provide new insights into the neurocognitive mechanisms of cognitive control and language processing in aphasia. Additionally, within the Chinese cultural context, our findings highlight the involvement of both Broca's area and the left DLPFC in integrating visual semantic information in poststroke aphasia patients. This addresses the prior underemphasis on Broca's area and underscores its unique role in visual language processing in Chinese. Additionally, fNIRS can serve as a valuable diagnostic tool. By assessing the hemodynamic response in different brain regions, fNIRS could assist clinicians in identifying early signs of neurocognitive impairments, particularly in populations with conditions like stroke, TBI, or neurodegenerative diseases. Furthermore, fNIRS can be used to monitor the effectiveness of rehabilitation therapies by comparing brain activity patterns before and after treatment. This real-time assessment allows clinicians to track changes in brain function, providing insights into the effectiveness of interventions and guiding adjustments in therapeutic strategies for optimal outcomes.

This study has several limitations that should be acknowledged. First, the relatively small sample size restricts the generalizability of our findings, especially given the heterogeneity within the poststroke aphasia population. To enhance robustness and external validity, future research should aim to recruit larger and more diverse cohorts. Additionally, this study did not include a wide representation of aphasia subtypes or account for lesion-specific effects, both of which are critical factors that could influence Stroop task performance and hemodynamic responses. These unaddressed variables may have introduced variability in brain activation patterns and behavioral outcomes. Moreover, our focus on hemodynamic responses may not fully capture the range of cognitive and neural mechanisms underlying task performance. Although the WAB was used for general classification, we did not perform detailed differentiation of aphasia subtypes or lesion locations. Given the heterogeneity of aphasia populations, performance likely varies significantly depending on these factors [69]. Future research should prioritize more precise stratification of participants and consider lesion-specific effects to gain a clearer understanding of the unique processing differences among aphasia subtypes. Such efforts could advance tailored clinical assessments and inform individualized intervention strategies, ultimately improving cognitive and language outcomes for this population.

## 5. Conclusion

This study used fNIRS to examine hemodynamic changes in poststroke aphasia patients during the Stroop task, revealing significant differences in blood flow compared to healthy controls. Patients with poststroke aphasia showed abnormal changes in hemoglobin concentrations, particularly in the left DLPFC and Broca's area, with notable alterations in HbO and DHb during incongruent conditions. These findings suggest that poststroke aphasia patients face difficulties in resolving cognitive conflicts, allocating attention, and integrating language. This is the first study to combine fNIRS with the Stroop task to gain deeper insights into the neural mechanisms underlying poststroke aphasia. Future research should explore different aphasia types and lesion locations in more detail. Based on these results, noninvasive brain modulation techniques such as rTMS and tDCS may offer potential for improving cognitive and language functions in poststroke aphasia patients by modulating brain activity, potentially enhancing cognitive efficiency and language recovery.

## Figures and Tables

**Table 1:** Results of *t*-test for the participants' ages.

Group	Number	Age (mean ± SD)	*t*
Aphasia	15	15	—
Control	54 ± 13	52 ± 17	0.36

**Table 2:** ANOVA results for chromophore (HbO, DHb, and tHb) levels across channels (34, 32, 36, and 50) in aphasia and control groups under neutral conditions.

Channel	Chromophore	Aphasia group	Control group	*F*_(1,28)_	*p*	Partial *η*^2^
34	HbO	0.05 ± 0.25	0.04 ± 0.13	0.001	0.975	0.000
	DHb	0.11 ± 0.28	0.07 ± 0.21	0.159	0.693	0.006
	tHb	0.16 ± 0.43	0.12 ± 0.27	0.084	0.774	0.003

32	HbO	0.06 ± 0.37	0.18 ± 0.46	0.695	0.412	0.024
	DHb	0.02 ± 0.17	−0.05 ± 0.24	0.894	0.352	0.031
	tHb	0.08 ± 0.52	0.14 ± 0.38	0.111	0.741	0.004

36	HbO	0.15 ± 0.32	0.10 ± 0.14	0.349	0.559	0.012
	DHb	0.01 ± 0.11	−0.02 ± 0.05	0.792	0.381	0.027
	tHb	0.16 ± 0.30	0.08 ± 0.16	0.861	0.361	0.030

50	HbO	0.04 ± 0.18	0.23 ± 0.17	9.294	0.005*⁣*^*∗∗*^	0.249
	DHb	−0.01 ± 0.18	−0.02 ± 0.16	0.031	0.862	0.001
	tHb	0.03 ± 0.29	0.21 ± 0.24	3.595	0.068	0.114

*⁣* ^*∗*^ *p* < 0.05, *⁣*^*∗∗*^*p* < 0.01.

**Table 3:** ANOVA results for chromophore (HbO, DHb, and tHb) levels across channels (34, 32, 36, and 50) in aphasia and control groups under congruent conditions.

Channel	Chromophore	Aphasia group	Control group	*F*_(1,28)_	*p*	Partial *η*^2^
34	HbO	−0.02 ± 0.14	0.01 ± 0.06	0.883	0.355	0.031
	DHb	0.07 ± 0.22	0.02 ± 0.08	0.743	0.396	0.026
	tHb	0.05 ± 0.27	0.03 ± 0.11	0.037	0.849	0.001

32	HbO	−0.03 ± 0.33	0.17 ± 0.44	2.001	0.168	0.067
	DHb	0.02 ± 0.20	−0.04 ± 0.13	0.757	0.392	0.026
	tHb	−0.02 ± 0.53	0.13 ± 0.43	0.712	0.406	0.025

36	HbO	0.09 ± 0.29	0.14 ± 0.30	0.212	0.649	0.008
	DHb	0.04 ± 0.08	−0.02 ± 0.08	5.629	0.025*⁣*^*∗*^	0.167
	tHb	0.14 ± 0.35	0.12 ± 0.22	0.028	0.868	0.001

50	HbO	0.01 ± 0.27	0.20 ± 0.28	3.620	0.067	0.114
	DHb	−0.03 ± 0.12	−0.12 ± 0.23	1.800	0.191	0.060
	tHb	−0.02 ± 0.24	0.08 ± 0.18	1.597	0.217	0.054

*⁣* ^*∗*^ *p* < 0.05, *⁣*^*∗∗*^*p* < 0.01.

**Table 4:** ANOVA results for chromophore (HbO, DHb, and tHb) levels across channels (34, 32, 36, and 50) in aphasia and control groups under incongruent conditions.

Channel	Chromophore	Aphasia group	Control group	*F*_ (1,28)_	*p*	Partial *η*^2^
34	HbO	0.04 ± 0.17	−0.07 ± 0.10	4.392	0.045*⁣*^*∗*^	0.136
	DHb	0.08 ± 0.21	−0.07 ± 0.19	4.407	0.045*⁣*^*∗*^	0.188
	tHb	0.12 ± 0.29	−0.14 ± 0.27	6.497	0.017*⁣*^*∗*^	0.161

32	HbO	−0.21 ± 0.47	0.17 ± 0.44	5.370	0.028*⁣*^*∗*^	0.153
	DHb	−0.01 ± 0.12	−0.01 ± 0.09	0.003	0.954	0.146
	tHb	−0.22 ± 0.55	0.16 ± 0.41	4.773	0.037*⁣*^*∗*^	0.004

36	HbO	0.03 ± 0.19	0.05 ± 0.12	0.099	0.756	0.270
	DHb	0.03 ± 0.06	−0.04 ± 0.05	10.347	0.003*⁣*^*∗∗*^	0.017
	tHb	0.06 ± 0.24	0.01 ± 0.11	0.475	0.496	0.163

50	HbO	−0.01 ± 0.22	0.16 ± 0.19	5.464	0.027*⁣*^*∗*^	0.005
	DHb	−0.03 ± 0.17	−0.05 ± 0.16	0.149	0.702	0.114
	tHb	−0.04 ± 0.26	0.12 ± 0.17	3.619	0.067	0.054

*⁣* ^*∗*^ *p* < 0.05, *⁣*^*∗∗*^*p* < 0.01.

## Data Availability

The data of fNIRS supporting the findings of this study are available at https://data.mendeley.com/datasets/3r8x3zh458/3. For more detailed information, please contact the corresponding author.

## Supporting Information

Supporting Information The supporting information includes descriptions of the results in Tables 2–4 and the scalp with MNI coordinates for the fNIRS data.
