# Supplementary material for: Unveiling Cognitive Interference: fNIRS Insights Into Poststroke Aphasia During Stroop Tasks
Source: Neural Plast. 2025 Mar 31;2025:1456201. doi: 10.1155/np/1456201 (PMC11976049; doi:10.1155/np/1456201)
Supplement: Supporting Information — The supporting information includes descriptions of the results in Tables 2–4 and the scalp with MNI coordinates for the fNIRS data. [file 1456201.f1.docx]

# Experimental Setup for Light Emission and Detection on the Scalp with MNI Coordinates of fNIRS


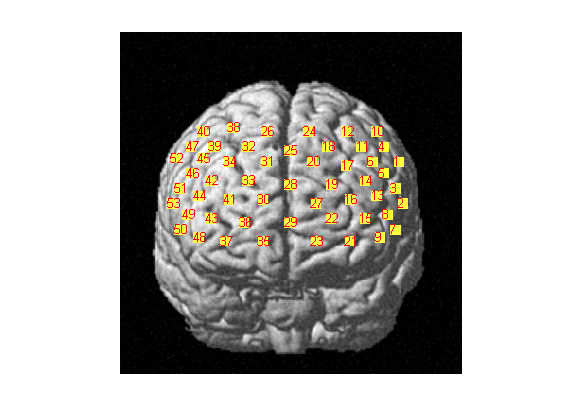


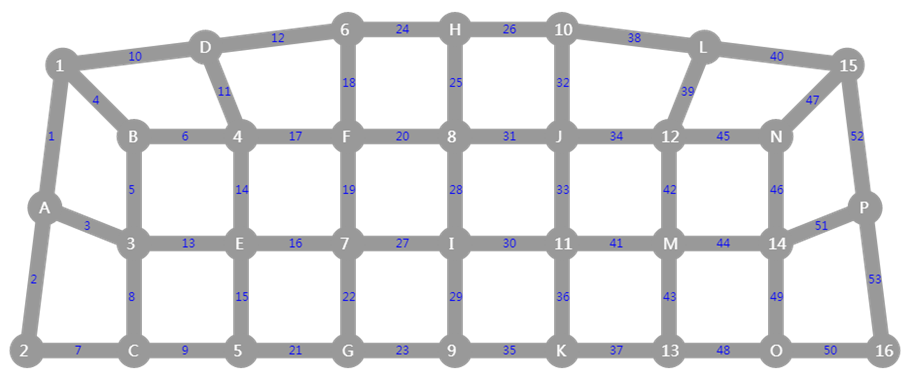


The wavelengths of the emitted light are 760 nm and 850 nm, with a frequency of 10 Hz. The distance between each emitter and detector is 3 cm. The space between the emitter and detector probes is a channel. Each probe was positioned on the forehead of the scalp. The above two images provided detail the MNI coordinates for each measurement channel.

**Table: Channel Mapping to Brodmann Areas**

| **Brodmann Area** | **Channels** |
| --- | --- |
| **Premotor Cortex and Supplementary Motor Cortex** | CH01, CH04, CH10, CH40, CH47, CH52 |
| **Broca's Area** | CH02, CH03, CH05, CH07, CH08, CH13, CH44, CH46, CH49, CH50, CH51, CH53 |
| **Dorsolateral Prefrontal Cortex** | CH06, CH09, CH11, CH14, CH17, CH18, CH20, CH25, CH31, CH32, CH34, CH39, CH42, CH45, CH48 |
| **Frontal eye field** | CH12, CH24, CH26, CH38 |
| **frontal lobe** | CH15, CH16, CH19, CH21, CH22, CH23, CH27, CH28, CH29, CH30, CH33, CH35, CH36, CH37, CH41, CH43 |

**Result**

Table 2 shows that, compared to the control group, the aphasia group exhibited significantly lower HbO levels in Channel 50 (Broca’s area) under neutral conditions (*p* < 0.01). Specific comparisons indicate that the mean HbO value in the aphasia group is significantly lower than that in the control group.

Table 3 demonstrated that under congruent conditions, there was a notable distinction (p < 0.05) in DHb levels within Channel 36 (frontal pole) between the groups sampled.A meticulous comparison of these variances revealed that the average measurement within the aphasia group exceeded that observed in the control group.

Table 4 presents the results of ANOVA comparing chromophore levels (HbO, DHb and tHb) between the aphasia group and the control group across different brain channels under incongruent Stroop task conditions.Channel 34: Significant differences were observed for all three chromophores. Specifically, the aphasia group exhibited higher levels of HbO, DHb, and tHb compared to the control group(*p* < 0.05).Channel 32: The aphasia group showed significantly lower levels of HbO and tHb compared to the control group (*p* < 0.05). No significant difference was found for DHb. Notably, this contrasting pattern of results is observed within the left DLPFC, highlighting an intriguing divergence in activation within this region.Channel 36: Only DHb showed a significant difference(*p* < 0.01), with the aphasia group having higher levels compared to the control group. No significant differences were found for HbO and tHb.Channel 50: HbO levels were significantly lower in the aphasia group compared to the control group(*p* < 0.05). There were no significant differences for DHb or tHb.For further illustration, Figure 4 displays the time course of changes in HbO, DHb, and tHb during the incongruent Stroop task for the two groups: the aphasia group and the control group. Data are averaged across all subjects within each group for Channel 34 (left dorsolateral prefrontal cortex) and Channel 50 (Broca's area).
